# Effect of a Flared Renal Stent on the Performance of Fenestrated Stent-Grafts at Rest and Exercise Conditions

**DOI:** 10.1177/1526602816651425

**Published:** 2016-05-25

**Authors:** Harkamaljot Kandail, Mohamad Hamady, Xiao Yun Xu

**Affiliations:** 1 Department of Chemical Engineering, Imperial College London, UK; 2 Department of Interventional Radiology, St Mary’s Hospital, Imperial College Healthcare NHS Trust, London, UK

**Keywords:** computational fluid dynamics, fenestrated stent-grafts, hemodynamics, renal stent, stent flaring

## Abstract

**Purpose:** To quantify the hemodynamic impact of a flared renal stent on the performance of fenestrated stent-grafts (FSGs) by analyzing flow patterns and wall shear stress–derived parameters in flared and nonflared FSGs in different physiologic scenarios. **Methods:** Hypothetical models of FSGs were created with and without flaring of the proximal portion of the renal stent. Flared FSGs with different dilation angles and protrusion lengths were examined, as well as a nonplanar flared FSG to account for lumbar curvature. Laminar and pulsatile blood flow was simulated by numerically solving Navier-Stokes equations. A physiologically realistic flow rate waveform was prescribed at the inlet, while downstream vasculature was modeled using a lumped parameter 3-element windkessel model. No slip boundary conditions were imposed at the FSG walls, which were assumed to be rigid. While resting simulations were performed on all the FSGs, exercise simulations were also performed on a flared FSG to quantify the effect of flaring in different physiologic scenarios. **Results:** For cycle-averaged inflow of 2.94 L/min (rest) and 4.63 L/min (exercise), 27% of blood flow was channeled into each renal branch at rest and 21% under exercise for all the flared FSGs examined. Although the renal flow waveform was not affected by flaring, flow within the flared FSGs was disturbed. This flow disturbance led to high endothelial cell activation potential (ECAP) values at the renal ostia for all the flared geometries. Reducing the dilation angle or protrusion length and exercise lowered the ECAP values for flared FSGs. **Conclusion:** Flaring of renal stents has a negligible effect on the time dependence of renal flow rate waveforms and can maintain sufficient renal perfusion at rest and exercise. Local flow patterns are, however, strongly dependent on renal flaring, which creates a local flow disturbance and may increase the thrombogenicity at the renal ostia. Smaller dilation angles, shorter protrusion lengths, and moderate lower limb exercise are likely to reduce the risk of thrombosis in flared geometries.

## Introduction

**Figure 1.**
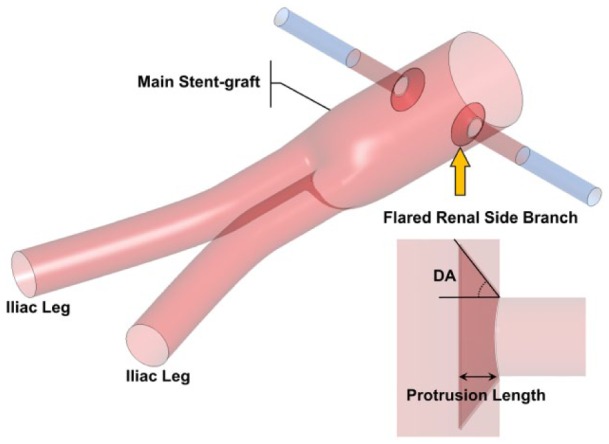
Schematic of the (flared) fenestrated stent-graft (FSG). The main stent-graft refers to the section of the FSG above the bifurcation, hosting the fenestrations. Flared renal stents are depicted with a yellow arrow, while the FSG is shown in red and extended renal arterial sections in blue. Definitions of DA and protrusion length are given in the lower right corner.

Minimally invasive fenestrated endovascular aneurysm repair (FEVAR) has become a common treatment procedure for abdominal aortic aneurysm (AAA) patients who were previously deemed unsuitable for EVAR due to either adverse aortic neck morphology or the presence of a suprarenal aneurysm. The fenestrated stent-grafts (FSGs) that are utilized in FEVAR are custom-made devices comprised of a main stent-graft, 2 iliac legs, and 2 renal side branches (Figure 1). The main stent-graft of FSGs has wire reinforced holes or fenestrations^1^ that are tailored to align with the ostia of renal arteries; through these fenestrations, renal stents are deployed to secure and seal the branch artery to the main stent-graft.

Theoretically, renal stents can be bare metal or covered stents. However, covered stents are increasingly used due to superior patency and reduced risk of intimal hyperplasia.^2,3^ These stents are secured in place by allowing a significant portion of the stent, typically between 3 and 5 mm, to protrude into the main stent-graft.^3–5^ The protruding section of the stent is then dilated using a balloon to form a funnel-shaped conduit for blood flow into the visceral arteries (Figure 1).^3^ This balloon dilation process is usually referred to as “flaring” by many clinicians and is necessary to develop an intimate contact between the visceral side branch and the fenestration.^1^

Even though FSGs show promising short to midterm clinical results,^6–8^ there is a pressing need to quantify their hemodynamic efficiency in order to assess their long-term durability. The use of computational fluid dynamics (CFD) through an interdisciplinary approach between engineering and medicine to quantify hemodynamics in FSGs has gained a lot of momentum in recent years.^9^ One reason for the increasing popularity of CFD simulations in evaluating the durability of FSGs is that they are cost-effective and reliable when used in conjunction with physiologically correct boundary conditions.

A number of CFD studies have been done on FSGs to evaluate their efficacy for FEVAR. For example, Avrahami et al,^10^ Georgakarakos et al,^11,12^ Kandail et al,^13,14^ Sun et al,^15^ and Sutalo et al,^16^ among others, performed CFD simulations on either patient-specific or ideal models of FSGs to shed light on hemodynamic conditions within the fenestrated endoprostheses. With the exception of Sun et al,^15^ most of these studies, however, neglected the effect of a flared renal stent. Sun et al^15^ examined the hemodynamic effect of flared fenestrated stent wires on renal blood flow in FSGs with bare renal stents. Their simulation results showed the effect to be negligible.

Since covered renal stent-grafts are frequently used clinically, it would be interesting to know the effect of flaring with covered renal stent-grafts. Another gap in the literature is that most studies were performed under resting conditions, and little is known about the effect of flaring under exercise conditions, which can increase cardiac output considerably, leading to different flow patterns.

The primary objective of this study was to address the unanswered questions that are of clinical importance: (1) the effect of flaring on renal flow waveform, (2) the risk for thrombosis in different FSGs, and finally (3) how FSGs perform under moderate lower limb exercise conditions. Answering these questions will aid surgeons and FSG manufacturers alike in predicting the likely outcome of FEVAR and thus optimize the procedure and FSG design.

## Methods

Performing CFD simulations is a multistage process, and defining 3-dimensional (3D) geometries comprising the fluid domain of interest is the first step. Once these geometries are constructed, they should then be subdivided into a fine numerical grid that serves as a template on which variables of interest (velocity and pressure) are obtained using numerical approaches, such as those based on the finite volume or finite element methods. These steps are elaborated in detail below.

### Geometry Construction

**Figure 2.**
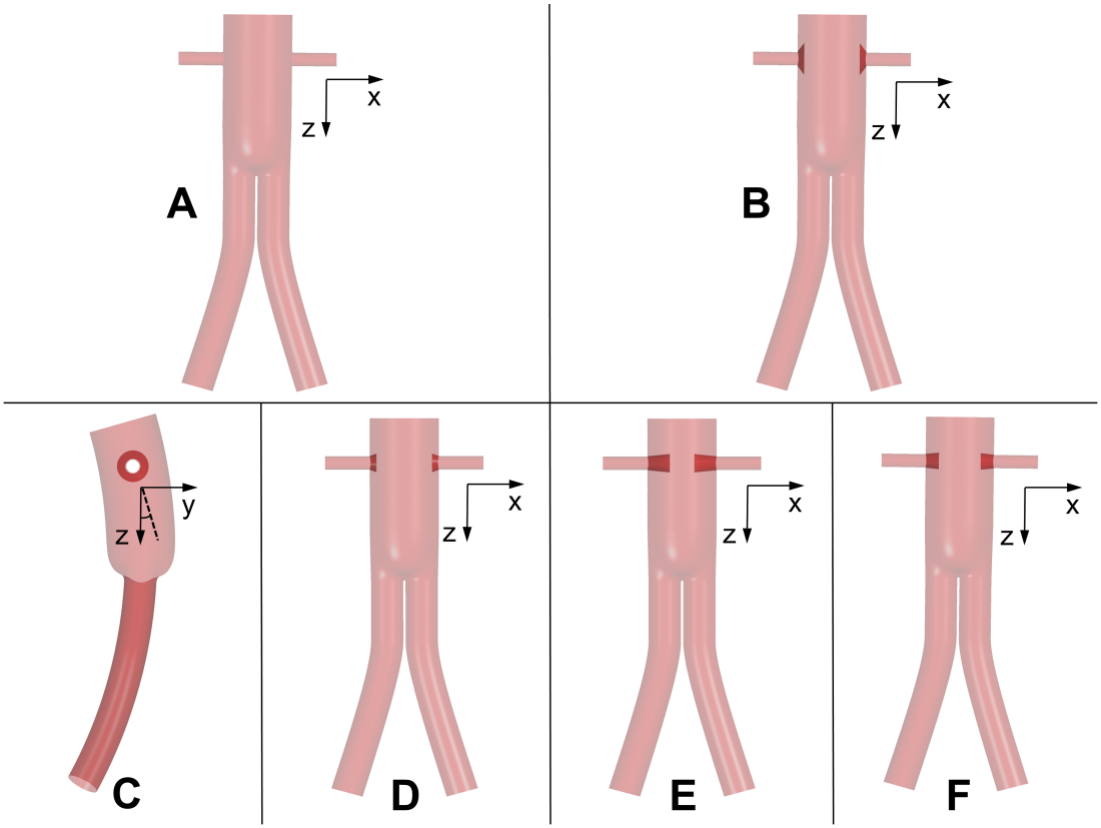
Schematic of all the analyzed geometries: (A) nonflared fenestrated stent-graft (FSG), (B) flared FSG type 1, (C) nonplanar FSG, (D) flared FSG type 2, (E) flared FSG type 3, and (F) flared FSG type 4. All are shown in the coronal plane except the nonplanar flared FSG, which is shown in the sagittal plane. Definition of anterior/posterior neck angle (APA) is also elaborated in panel C, where the solid black line is the *z*-axis in the sagittal plane and the dashed black line is the surface normal to the inlet; the APA is the angle between the two.

Hypothetical models of FSGs were constructed using SolidWorks (Dassault Systemes, Velizy, France), based on clinical findings of Sun et al^4^ and Sun,^5^ who reported that out of 27 visceral stent-grafts, 15% were of circular appearance with flaring, while 37% were circular with little flaring effects. The renal stent-grafts protruded into the main stent-graft with lengths ranging from 2.1 to 17.7 mm. With these data in mind, various models of FSGs were constructed and subdivided into 6 groups, each representing a specific morphology. As shown in Figure 2, these were (1) nonflared FSG, (2) flared FSG type 1, (3) nonplanar flared FSG, (4) flared FSG type 2, (5) flared FSG type 3, and (6) flared FSG type 4. Nonflared FSGs served as a reference case against which the results from other geometries were compared.

Dilation angles (DA; defined in Figure 1) due to flaring in all the studied FSGs, along with protrusion lengths of the renal stents into the main stent-graft, are summarized in Table 1. Three DAs were considered in this study, representing various extents of flaring, with larger DAs corresponding to higher extent of flaring (eg, flared FSG type 1). Theoretically, flared FSG type 1 represented the configuration where there is an intimate contact between the renal stent and the fenestration, while flared FSG types 3 and 4 were the least expected configurations. However, all these configurations are regularly seen in postoperative scans.^4,5^

**Table 1.** Dilation Angles Due to Flaring and Protrusion Lengths of the Renal Stents Into the Main Stent-Graft for All the Analyzed Geometries.

	Dilation Angles, deg^a^	Protrusion Length, mm
Flared FSG type 1	53.13	3
Flared FSG type 2	18.43	3
Flared FSG type 3	5.71	10
Flared FSG type 4	5.71	6
Nonplanar FSG	53.13	3

Abbreviation: FSG, fenestrated stent-graft.

a Definition is given in Figure 1.

It was also necessary to include nonplanarity in flared FSGs. Because the abdominal aorta rests on the curved lumbar vertebrae, FSGs tend to follow the natural curvature and nonplanarity of the vertebral column when they are deployed. Lumbar curvature is represented by an anterior/posterior neck angle (APA) of 20° based on previous studies^13,17^ (Figure 2), where APA is defined as the angle between the *z*-axis and the surface normal of the inlet in the sagittal plane.

Key dimensions of all the stent-graft models are: 31-mm inlet diameter, 14.5-mm iliac diameter, and 6-mm renal side branch diameters. As shown in Figure 1, renal branches were artificially extended by 30 mm in length to ensure that the model outlets were sufficiently far from regions of disturbance or any potential recirculation zones. It is worth noting that exercise simulations were performed only on the flared FSG type 1.

### Grid Generation

The 3D hypothetical models were discretized into fine unstructured meshes comprised of tetrahedral and prism elements using ANSYS ICEM CFD (ANSYS, Canonsburg, PA, USA). A hybrid mesh of tetrahedral elements in the core region and prism elements in the sheared boundary layer was adopted to allow better control of the near wall mesh resolution, which is important for accurate prediction of hemodynamic wall parameters. Local mesh refinement was performed using the curvature and proximity based refinement function in ANSYS ICEM CFD. The value for edge criterion was set as 0.05 to ensure that the mesh propagated through tight corners all the way to terminal curves. This was especially important when meshing regions between the flares and the stent-graft walls. Additionally, grid independence tests were carried out starting with meshes containing 500,000 elements until the predicted velocity fields and time-averaged wall shear stress (TAWSS) differed by <2% between the adopted mesh and a much finer mesh. The final computational simulations were performed on grids with around 6 million elements, with the near wall prismatic cell heights varying in the range of 0.05 to 0.19 mm.

### Computational Simulations

Laminar and pulsatile blood flows were simulated by numerically solving Navier-Stokes equations, which state that for a given isothermal system, mass and momentum are conserved. Blood was treated as an incompressible Newtonian fluid with a density of 1060 kg/m^3^ and viscosity of 0.004 Pa·s.

#### Boundary conditions for resting scenarios

**Figure 3.**
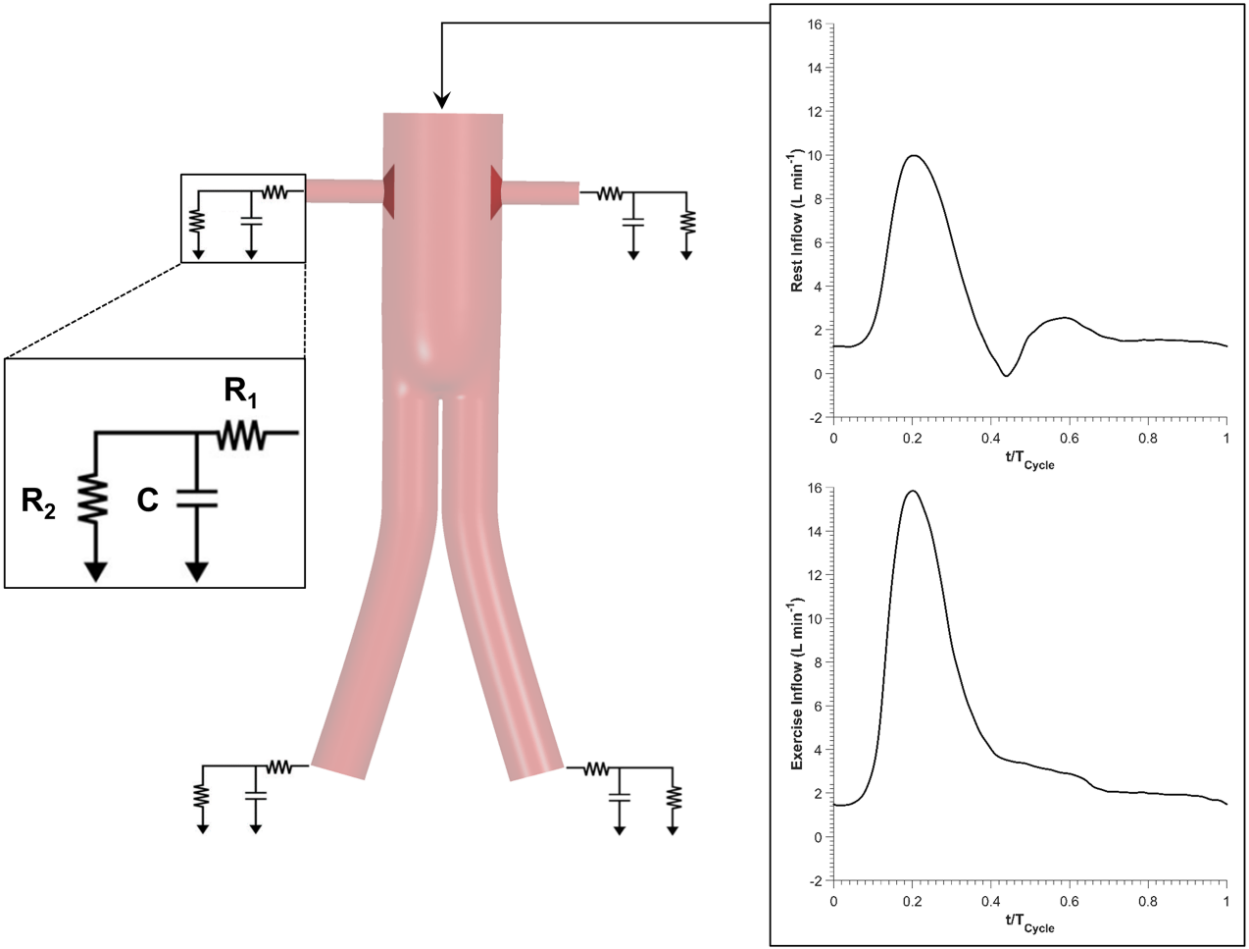
Detailed schematic of the computational model utilized in this study. Flow rate waveforms corresponding to rest and exercise conditions were prescribed at the inlet, while the vasculature distal to the fenestrated stent-graft (FSG) was modeled using a lumped parameter 3-element windkessel model.

In order to produce clinically relevant results, physiologically realistic boundary conditions were employed: (1) in vivo measured resting volumetric flow rate waveform^18^ at the inlet and (2) coupling of each outlet to a 3-element windkessel model (3-EWM). A schematic illustration of the computational model and the applied inflow waveforms can be found in Figure 3. The parameters of 3-EWM, that is, proximal resistance (R_1_), distal resistance (R_2_), and compliance (C) of the downstream vasculature, were fine tuned for nonflared FSGs to match the maximum, minimum, and cycle-averaged aortic blood pressures of 135, 60, and 86 mm Hg, respectively. As reported by Sonesson et al,^19^ these pressure values are representative of AAA patients. The same set of parameters was utilized for all the other geometries to facilitate direct comparison of results. The stent-graft walls were assumed to be rigid where no slip conditions were applied.

#### Boundary conditions for exercise scenarios

Using the approach proposed by Les et al,^18^ boundary conditions applied in resting scenarios were scaled appropriately to simulate moderate lower limb exercise. Heart rate was increased by 50%, equivalent to increasing beats per minute from 63 (rest) to 95 (exercise). Since cardiac output also increases during moderate exercise, cycle-averaged inlet flow was increased by 1.57 fold from 2.94 to 4.63 L/min. Exercise inlet flow waveform was antegrade throughout the cardiac cycle, that is, no backflow. Montain et al^20^ reported that even though systolic blood pressure increased considerably during moderate lower limb exercise, diastolic blood pressure remained about the same. Therefore, exercise parameters R_1_, R_2_, and C were altered as such to satisfy the observations of Montain et al^20^ while at the same time mimicking increased infrarenal blood flow. As a result, distal renal resistance was increased by 10.31% and distal iliac resistance was decreased by 36.11% from the resting conditions. Compliance of the renal arteries decreased while iliac compliance increased during exercise. As before, stent-graft walls were assumed to be rigid where no slip conditions were applied.

The aforementioned boundary conditions were implemented in ANSYS CFX through FORTRAN subroutines. A uniform time-step of 0.001 seconds was adopted while convergence criterion based on root mean square residual was set to be 1×10^−6^. Each simulation was performed for 3 cardiac cycles in order to achieve a periodic solution.

### Evaluation Objectives

The performance of the simulated FSGs with different configurations and physiologic scenarios was evaluated in terms of flow rate waveform in the renal arteries, flow patterns and structure in the main stent-graft, wall shear stress–related indices, and finally, displacement forces acting on the FSGs.

To assess the effectiveness of FSGs with and without flaring of the renal stent, it is essential to understand how they affect the flow to renal arteries. This is important because if renal perfusion falls below a certain threshold, usually 15% to 30% of the aortic inflow^21^ under resting conditions, serious complications may ensue. It is worth noting that aortic inflow refers to the flow distal to the superior mesenteric artery (SMA). Flow patterns at the entrance to the renal branches and around the renal ostia are displayed as instantaneous velocity streamlines.

In order to examine the influence of flaring on flow disturbances around the renal ostium, vortical structures in each FSG geometry were compared at the point of maximum flow deceleration. Vortical structures were evaluated using the lambda-2 criterion,^22^ which is a mathematical algorithm capable of identifying 3D vortices from the local velocity fields.

Wall shear stress–related indices, namely TAWSS and oscillatory shear index (OSI), have been commonly used to identify regions at higher risk of wall thickening and thrombus formation.^23–25^ OSI is derived from TAWSS and has a value between 0 (for totally unidirectional flow) and 0.5 (for oscillatory flow with equal antegrade and retrograde components). It has been found that regions that are prone to thrombosis typically experience high OSI and low TAWSS. Based on this observation, Di Achille et al^26^ proposed a new metric, endothelial cell activation potential (ECAP), defined as the ratio of OSI to normalized TAWSS, as a surrogate to identify regions that might be at an elevated risk of thrombosis. In this study, TAWSS was normalized by the spatial average value of TAWSS at the most proximal part of the corresponding geometry. Since ECAP is capable of identifying regions exposed simultaneously to high OSI and low TAWSS, it was calculated for all the analyzed FSGs. It is worth mentioning that the 99th percentile values of ECAP are presented instead of the maximum nodal values to eliminate spuriously high values at isolated spots.

The magnitude and direction of displacement forces experienced by all the FSGs were calculated by integrating the traction vectors (pressure and wall shear stress) over the entire surface of the FSG. As reported previously on patient-specific FSGs,^13^ the time course of displacement forces follows the pressure waveform very closely.

## Results

### Flow in the Renal Arteries

**Figure 4.**
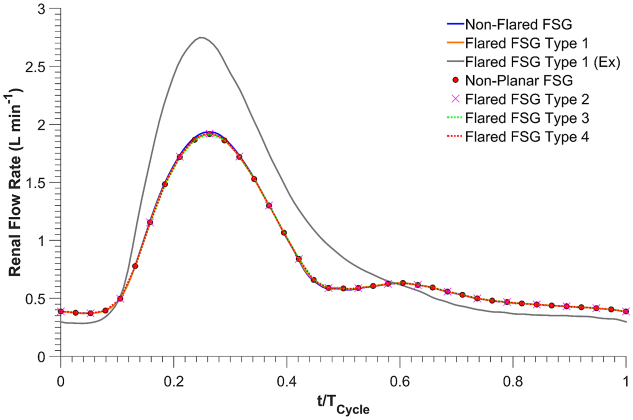
Renal flow rate waveforms for all the analyzed fenestrated stent-grafts (FSGs); Ex refers to during exercise.

As displayed in Figure 4, under resting conditions, flow in each renal branch for nonflared and all flared FSG configurations was almost identical, with a cycle-averaged flow rate of ~0.80 L/min, accounting for 27.06% of the inflow. Cycle-averaged infrarenal flow was 1.35 L/min, accounting for 45.88% of the inflow.

Under exercise conditions for flared FSG type 1, peak systolic flow in each renal branch increased by 43.47%, from 1.92 L/min (resting conditions) to 2.75 L/min (exercise conditions). During the relaxation phase of the cardiac cycle, however, flow in each renal branch was lower than the resting case. Renal exercise outflow for flared FSG type 1 at terminal diastole was 0.28 L/min, ie, a reduction of 23.31% from rest (resting terminal diastolic renal outflow for the same geometry was 0.37 L/min). Overall, the cycle-averaged flow in renal branches of flared FSG type 1 was 22.44% higher during exercise at 0.97 L/min, accounting for 21.01% of the inflow. It is also evident from Figure 4 that renal blood flow was always antegrade under both resting and exercise conditions. Cycle-averaged infrarenal flow during exercise was 2.69 L/min, accounting for 57.98% of the inflow.

**Figure 5.**
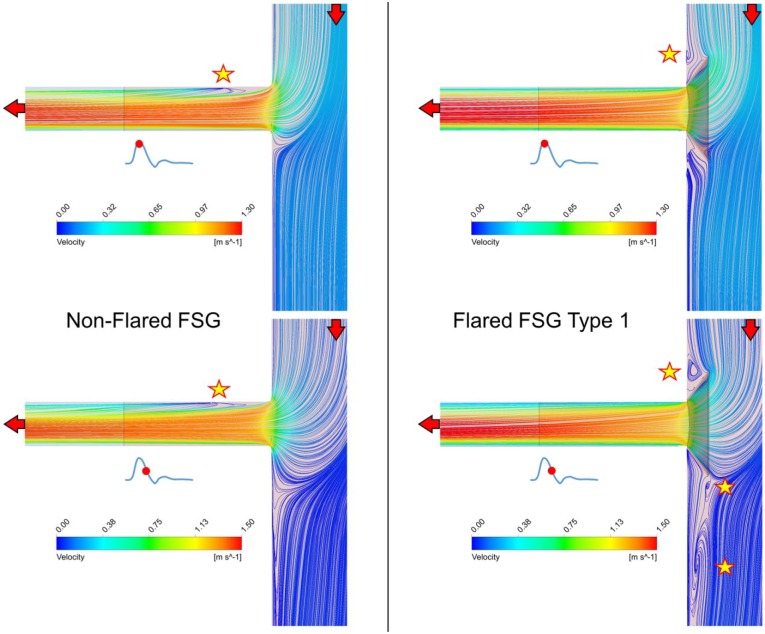
Instantaneous velocity streamlines plotted on the cut plane dividing the fenestrated stent-graft (FSG) models in 2 equal parts in the coronal plane. Red arrows denote the direction of the blood flow, while yellow stars depict the location of flow recirculation zones.

Flow velocity streamlines at the entrance to the renal branches and around the renal ostia are displayed in the coronal plane for nonflared FSGs and flared FSG type 1 (Figure 5). It can be seen that at peak systole (T1), there was one flow recirculation zone in the renal side branch of nonflared FSGs (as highlighted by a yellow star in Figure 5), but no recirculation was found in the renal branch in flared FSG type 1. There was, however, one recirculation zone above the renal ostium in the flared FSG type 1. At maximum flow deceleration (T2), the flow pattern in nonflared FSGs remained similar to that at T1, ie, a recirculation zone was still present at the same location in the renal branch. However, 3 recirculation zones were observed in the flared FSG type 1, one just above the renal ostium and two below the renal ostium. Similar flow patterns were observed for all the other flared geometries, but the extent of flow disturbance varied.

**Figure 6.**
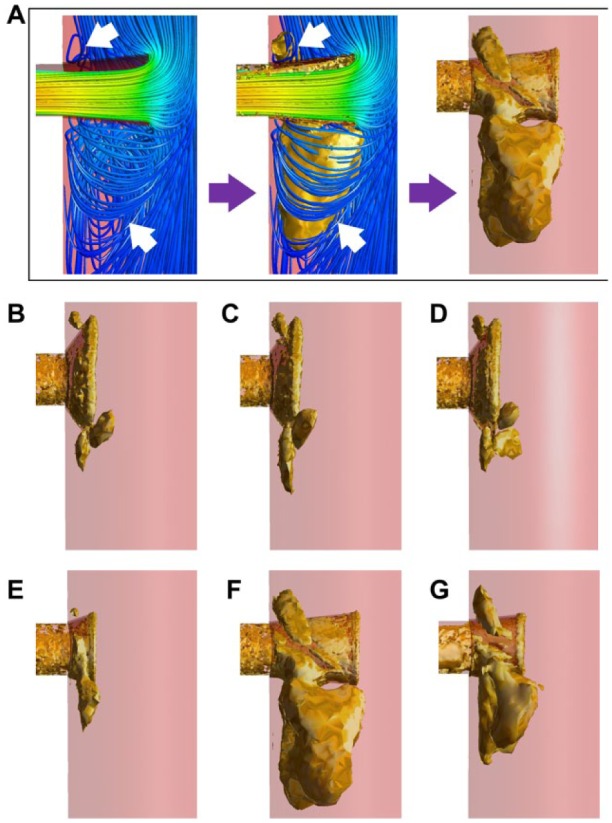
Vortical structures in all the studied geometries at the time point of maximum flow deceleration [t=0.3 seconds (rest) and 0.18 seconds (exercise)]. (A) Definition of the vortical structures plotted using the lambda-2 criterion. Disturbed flow or vortices found below the renal flares (arrows in left panel) and the golden surface (arrows in middle panel) engulf the entire vortex core region, thus representing the vortical structure (right panel). (B) Flared FSG type 1, (C) flared FSG type 1 (exercise conditions), (D) nonplanar flared FSG, (E) flared FSG type 2, (F) flared FSG type 3, and (G) flared FSG type 4. FSG, fenestrated stent-graft.

The 3D vortices depicting flow disturbances around the renal ostium are displayed as golden colored isosurfaces in Figure 6, encompassing the entire vortex core region (the definition of the lambda-2 criterion is also visually illustrated in Figure 6A). It can be seen that disturbed flows (or vortices) were observed in all flared geometries at the entrance to the renal branches. No vortical structures were observed in nonflared FSGs at the renal ostium; however, they were observed in all flared FSG configurations (Figure 6B-G). Similar vortical structures were observed in flared FSG type 1 (rest and exercise conditions) and nonplanar flared FSGs (all had a DA of 53.13° and a protrusion length of 3 mm), that is, small vortical structures above the flaring and much larger vortical structures below the flaring. Relatively smaller vortical structures were observed in flared FSG type 2, which had a DA of 18.43° and a protrusion length of 3 mm, with one small vortical structure above and one below the flaring. The largest vortical structures, however, were found in flared FSG type 3 with a DA of 5.71° and a protrusion length of 10 mm, while the second largest vortical structures were observed in FSG type 4, which also had a DA of 5.71° but protrusion length of 6 mm.

### Wall Shear Stress–Related Indices

**Figure 7.**
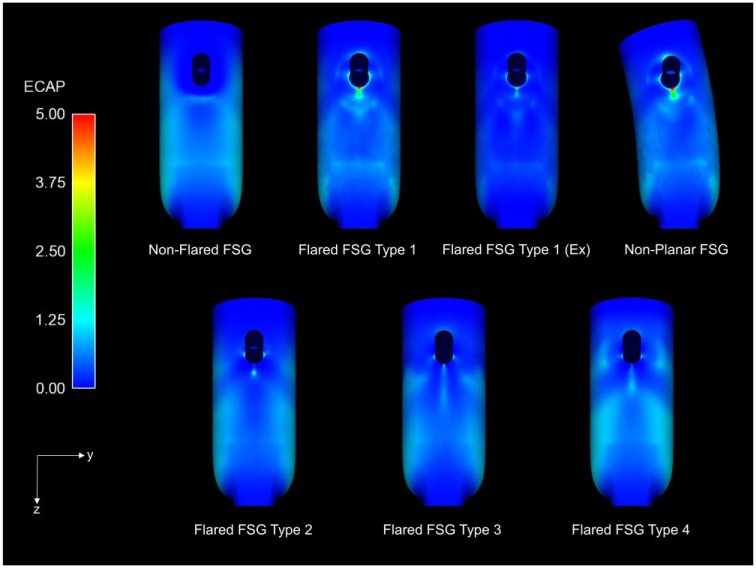
Endothelial cell activation potential (ECAP) contours for all the analyzed fenestrated stent-grafts (FSGs) in the sagittal plane. High ECAP values for all the flared geometries can be seen concentrated at the renal ostia. Ex refers to during exercise, while nonplanar FSG refers to nonplanar flared FSG.

Thrombus formation was not simulated directly in this study, but ECAP values were used as a surrogate to identify regions at an elevated risk of thrombosis. The ECAP values for all the analyzed FSGs are presented in Figure 7 (sagittal plane), but since ECAP values >5 are displayed in red, the 99th percentile values of ECAP for all the FSGs are summarized in Table 2. All flared FSGs had a small region of very high ECAP at the renal ostia, especially in flared FSG type 1 (rest and nonplanar), while ECAP values at the renal ostia in nonflared FSG were negligible. It is also clear that flared FSG type 1 under exercise condition had lower ECAP values than FSG type 1 at rest. Comparison of all flared FSGs at resting condition suggested that flared FSG type 2 had a lower ECAP value than the other configurations.

**Table 2.** Endothelial Cell Activation Potential (ECAP) 99th Percentile Values for All the Analyzed Fenestrated Stent-Grafts (FSGs).^a^

	ECAP 99th Percentile Values	Location
Nonflared FSG	1.47	Suprailiac bifurcation
Flared FSG type 1	3.29	Renal ostia
Flared FSG type 1 (exercise)	0.98	Renal ostia
Nonplanar FSG	3.56	Renal ostia
Flared FSG type 2	0.69	Renal ostia
Flared FSG type 3	1.02	Renal ostia
Flared FSG type 4	0.90	Renal ostia

a Locations of these values on the surface of the FSGs are shown in Figure 7. It is worth noting that in contrast to flared FSGs, ECAP values in the nonflared FSGs were ~0 at the renal ostia, and the 99th percentile value was observed at the suprailiac bifurcation in the coronal plane.

### Displacement Forces Acting on the FSG

**Figure 8.**
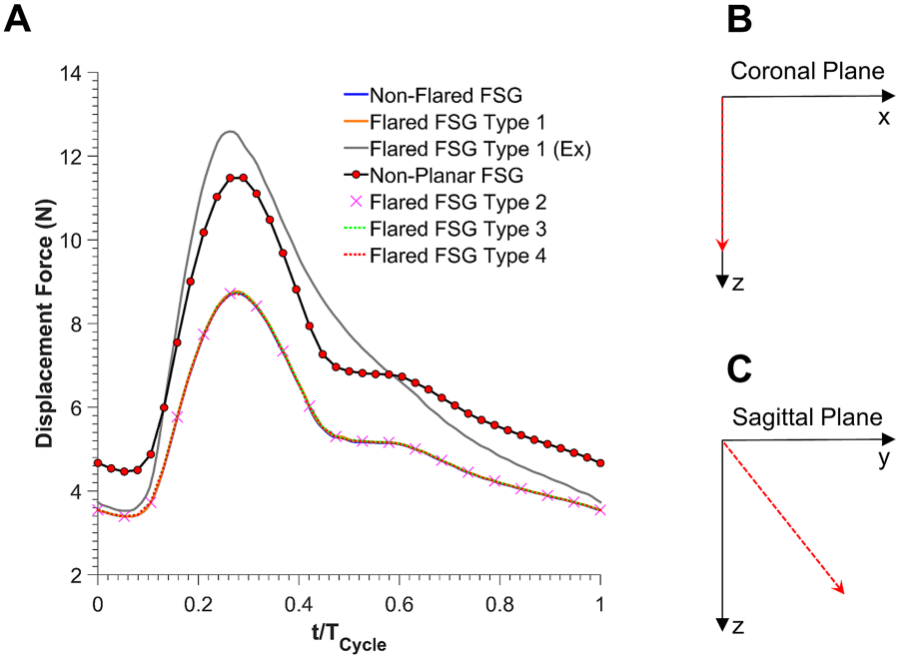
(A) Magnitude of the resultant displacement forces experienced by all the fenestrated stent-grafts (FSGs). (B) Direction of the displacement force acting on all the planar FSGs is shown by a red dashed arrow in the coronal plane. Angles with *x* and *z* were 90°, respectively, and this angle was constant over the entire cardiac cycle. (C) Direction of the displacement force acting on the nonplanar flared FSG is shown by red dashed arrow in the sagittal plane. Angles with *y* and *z* were 51° and 39°, respectively, and these angles were constant over the entire cardiac cycle. Ex refers to during exercise.

The magnitude and direction of displacement forces experienced by all the FSGs are summarized in Figure 8. Wall shear effects were <0.2% in all the cases. Both flared and nonflared FSGs experienced an equal cycle-averaged displacement force of 5.27 N. Nonplanarity increased the magnitude of displacement forces, and the cycle-averaged displacement force for nonplanar flared FSGs increased from 5.27 to 6.94 N. Exercise also had a profound effect on the displacement forces, with the maximum displacement force for flared FSGs under exercise conditions being significantly higher (12.59 N), which resulted in a high cycle-averaged displacement force (6.94 N).

All the planar FSGs experienced displacement forces acting vertically downward (Figure 8B) in the coronal plane. Nonplanar flared FSGs with an APA of 20°, however, experienced displacement forces acting at an angle of 51° with the *y*-axis and 39° with the *z*-axis in the sagittal plane (Figure 8C), and these directions did not change during a cardiac cycle.

## Discussion

The use of FSG in FEVAR has allowed a wider cohort of patients to be treated with this minimally invasive procedure. However, there are certain questions related to hemodynamics that still need to be answered from the design point of view in order to assess the long-term reliability and efficacy of these stent-grafts. This study employed CFD simulations with varying geometries and physiologic conditions to address one of these important questions, namely, does renal flaring affect the performance of FSGs as compared to their nonflared counterparts?

Under resting conditions (Figure 4), flaring of renal stents had a negligible effect on the renal flow waveform, with all FSGs having the same cycle-averaged flow rate. Exercise increased the flow to each renal artery (with flaring) by 21% due to increased infra-SMA flow during the systolic phase. It was also clear that flow in the flared renal branches was antegrade throughout the cardiac cycle at both resting and exercise conditions. These flow characteristics are crucial for maintaining normal renal function. The respective percentage renal flows during rest and exercise are in line with the results of Tang et al,^27^ who also reported that while exercise increased systolic renal flow, diastolic renal blood flow was closely matched to its resting value. Since under normal physiologic conditions, flow split to each renal artery is between 20% and 30% of the infra-SMA flow,^18,21,27–29^ it can be deduced from Figure 4 that flaring the renal stent does not affect blood perfusion to renal arteries at rest or during exercise.

Even though renal flow waveforms were not affected by flaring, spatial flow patterns and wall shear stress–related indices were altered. As highlighted in Figure 5, at peak systole there was a flow recirculation zone at the entrance of the renal side branch for nonflared FSGs due to the sudden change in flow direction commonly observed at junctions and bifurcations. In the case of flared FSGs, however, no recirculation zones were found within the renal side branch because the funnel-shaped flared stent guided the flow smoothly from the main stent-graft to the renal side branch. Though flaring improved the spatial flow patterns within the renal side branches, it caused additional disturbed flow within the main stent-graft right at the renal ostia. As shown in Figure 5, flared FSGs had flow recirculation zones that became evident especially during diastole, when 2 recirculation zones were observed just above and below the renal flares. Therefore, Figure 5 depicts clearly that even though flaring improved flow patterns within the renal side branch, it caused flow disturbance at the renal ostia outside the flared stent.

The extent of flow disturbance caused by flaring is influenced by DA and protrusion length. Figure 6 demonstrated that flared FSG type 2 with DA of 18.43° and a 3-mm protrusion length had the smallest vortical structures of all the flared geometries. As DA increased, the size and number of vortical structures also increased as shown in the case of flared FSG type 1 (rest and exercise) and nonplanar FSGs. The most drastic effect was, however, observed in flared FSG type 3, which had a DA of 5.71° and 10-mm protrusion length, where the largest vortical structure was observed. With the same DA but a shorter protrusion length, flared FSG type 4 had vortical structures that were smaller than those in flared FSG type 3 but larger than the ones observed in flared FSG type 2. These results demonstrated a strong dependence of vortical structures on the protrusion length of the renal stent into the main stent-graft, with longer protrusion leading to larger vortical structures.

The most distinct difference between flared and nonflared FSGs is summarized in Figure 7. As seen in the sagittal plane, due to flaring, the renal ostia in all the flared geometries were exposed to higher levels of ECAP because of the highly oscillatory nature of the flow recirculation zones above and below the renal flares and the slow flow within the recirculation zones (Figure 5). Di Achille et al^26^ reported that areas with normalized ECAP values >5 correlated very well with locations of thrombus in AAA patients; therefore, high values of ECAP may correspond to an elevated risk of thrombosis. The variations in the 99th percentile values of ECAP (Table 2) at the renal ostia of flared FSGs was greatest for the nonplanar flared FSGs, which suggests that flaring the renal stent might increase the risk of thrombosis at the renal ostia compared to nonflared FSGs.

It is also interesting to note that the 99th percentile ECAP values varied with DA, which indicates that a larger DA leads to higher maximum ECAP and thus increased susceptibility to thrombosis. Although the flared FSG type 3 had the lowest DA of all the analyzed geometries, it also had the largest protrusion length, which might be why the 99th percentile ECAP values for flared FSG type 3 were higher than that of flared FSG type 2. Results for flared FSG type 4 further confirmed that longer protrusion would lead to higher ECAP values (thus higher risk of thrombosis). Based on these findings, it can be deduced that for a fixed protrusion length (flared FSG type 1 and type 2), a smaller DA will result in lower ECAP values. Likewise, for a fixed DA (flared FSG types 3 and 4), increasing the protrusion length will also lead to higher ECAP values. These findings and observations are consistent with the vortical structures seen in Figure 6. It is worth noting that moderate exercise can reduce the 99th percentile ECAP values by 70%; moreover, the region of high to moderate ECAP was more concentrated and focal, implying that moderate exercise may improve the durability of flared FSGs by mitigating the thrombosis risk at the renal ostia.

As blood flows through the stent-grafts, it exerts pressure and wall shear stress on its surfaces.^30^ It is important to quantify these forces because they are responsible for device migration and can lead to future complications, such as occlusion and/or endoleaks. Flaring did not alter the magnitude of displacement forces (Figure 8). Nonplanarity, however, had a profound influence on displacement forces, as demonstrated by a 32% increase in the resting cycle-averaged displacement force compared with the planar configuration. This effect has also been reported by other researchers.^13,17,31^ When mild exercise conditions were simulated for flared FSGs, the minimum displacement force was very comparable to resting conditions even though the maximum displacement force increased. This was because pressure fields were significantly affected during exercise, with peak pressure rising considerably while the minimum aortic pressure remained about the same. In their experimental study, Melas et al^32^ reported that the displacement force required to dislocate the proximal portion of planar stent-grafts (with hooks or barbs) by >20 mm was 26.97 N for self-expanding stent-grafts and 32.45 N for balloon-expandable devices. If the stent-grafts were not equipped with hooks or barbs, the displacement force required to produce the same dislocation reduced to 13.58 N for self-expanding stent-grafts and 14.72 N for balloon-expandable devices. Comparing the cycle-averaged displacement forces for all the planar FSGs with these experimental data, it can be deduced that these devices are at a low risk of distal migration. Rahmani et al^33^ reported that for nonplanar stent-grafts with an APA of 20°, the force required to dislocate the proximal section of balloon-expandable stent-grafts (with hooks and barbs) was also ~32 N. The cycle-averaged displacement force for nonplanar FSG in this numerical study was found to be 6.94 N, indicating that even the nonplanar FSG would be at low risk of distal migration.

### Limitations

The computational models employed in this study have a number of limitations. First, the stent-graft geometries included in this study were idealized rather than patient-specific. Numerical simulations of patient-specific flared FSG geometries should be performed in the future to complement the findings from this study.

Second, due to flaring, the protruded section of the renal stent can deform into any shape ranging from circular (as assumed here) to elliptical or irregular. The assumption of a circular stent-graft was made based on the study of Sun et al,^4^ who examined 27 fenestrations and found that 52% were circular. Third, only covered stents were considered, and these were assumed to be smooth without explicit modeling of stent wires at the end. Stent wires typically have a diameter between 0.3 and 0.5 mm, which can alter local wall shear stress considerably. This is a serious limitation and will be considered in depth in future studies.

Fourth, as reported by Draney et al^34^ and Ullery et al,^35^ renal arteries can bend by as much as 21° during the respiratory cycle, however, these effects were ignored in the present study. Finally, vessel walls were assumed to be rigid. Previous studies^13,36^ have demonstrated that nitinol wires (used in most stents) and graft materials (typically made of expanded polytetrafluoroethylene or polyethylene terephthalate) are very stiff, with a large Young’s modulus, thus compliance effects are expected to be minor.

## Conclusion

While flaring does not compromise flow to the renal arteries under rest and exercise conditions, it does expose the renal ostia to elevated levels of ECAP, making these regions potentially susceptible to thrombus formation. Nevertheless, the level of ECAP can be lowered considerably by reducing the dilation angle or protrusion length or through moderate lower limb exercise, which may help improve the durability of flared FSG. Even though exercise and nonplanarity increase the magnitude of displacement forces, the rise observed in this study was not significant enough to indicate a risk of device migration.
